# Supplementary figures and images for: Using a Deliberative Poll on breast cancer screening to assess and improve the decision quality of laypeople
Source: PLoS One. 2021 Oct 21;16(10):e0258869. doi: 10.1371/journal.pone.0258869 (PMC8530304; doi:10.1371/journal.pone.0258869)

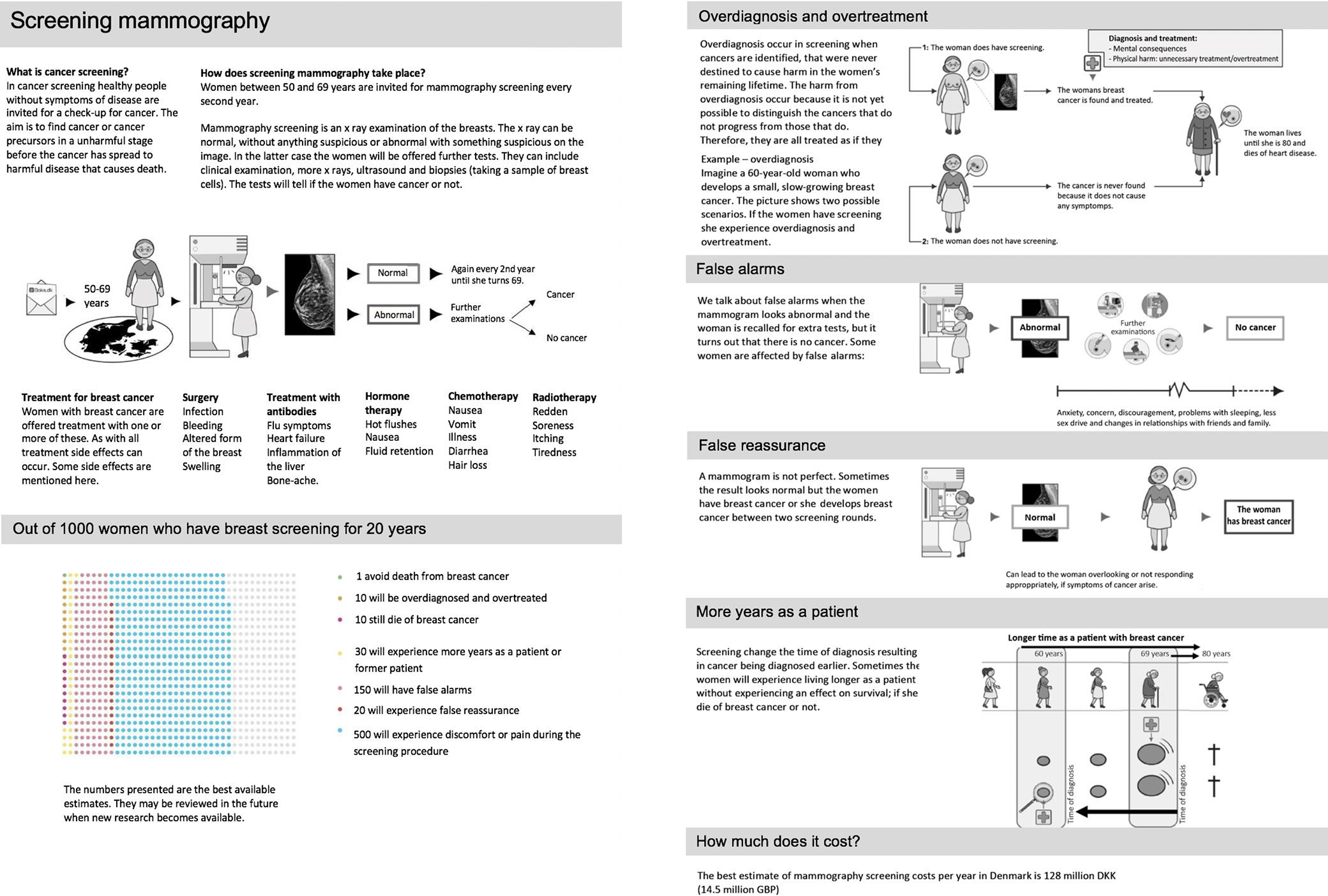

Supplement: S1 Fig — (TIF) [file pone.0258869.s001.tif]

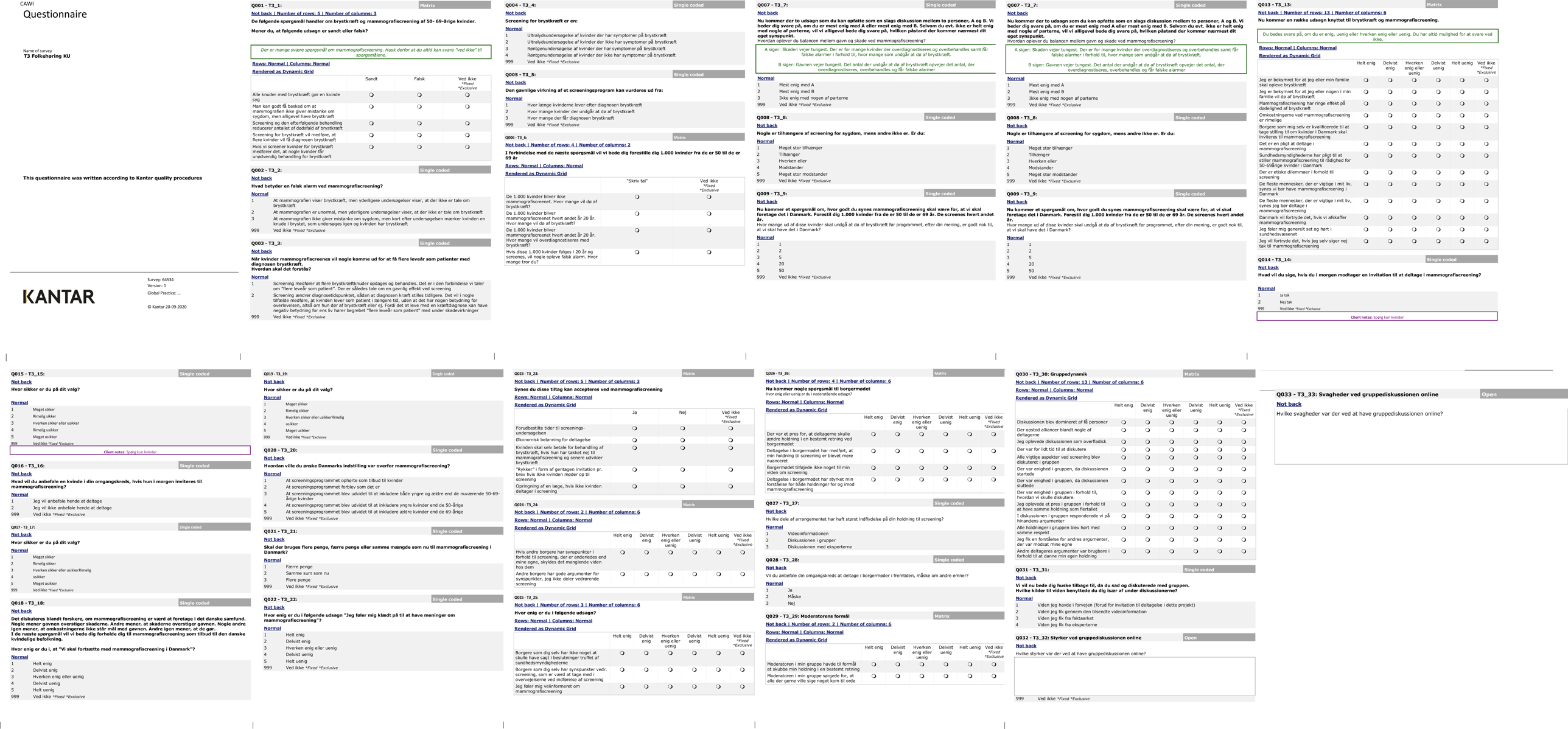

Supplement: S2 Fig — (TIF) [file pone.0258869.s002.tif]

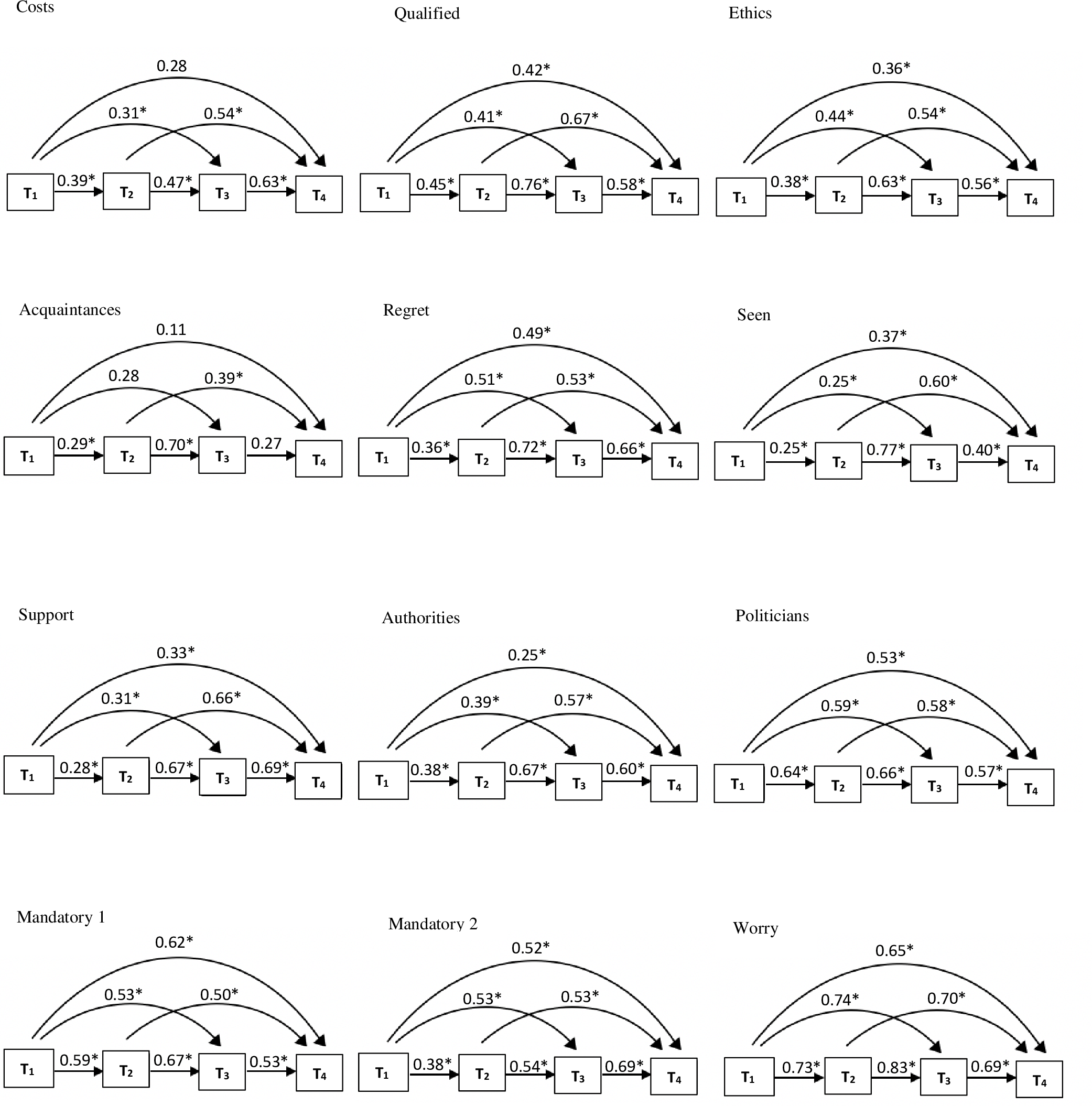

Supplement: S3 Fig — Note: The figure shows the intercorrelations within the opinions over time. The more a correlation approximates one the more the answers from the two timepoints are in line. n varies: between 89 and 85. T1 = inquiry time point 1 (recruitment), T2 = inquiry time point 2 (after video information), T3 = inquiry time point 3 (after deliberation), T4 = inquiry time point 4 (one month after the citizens’ assembly). (TIF) [file pone.0258869.s003.tif]

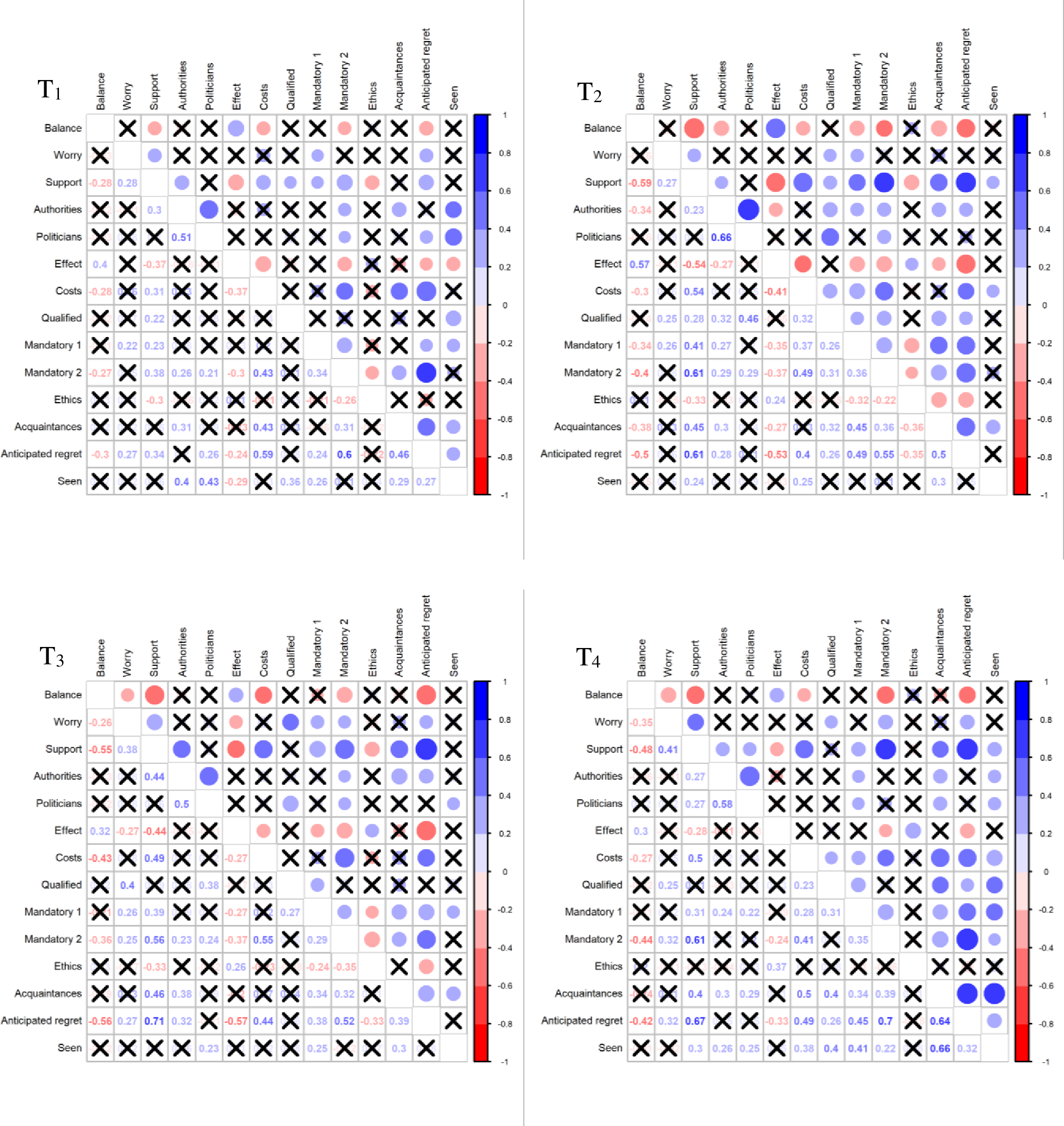

Supplement: S4 Fig — Note: The figure shows the internal correlation between opinion items at four time points. A correlation of 1.00 (or -1) indicates a perfectly consistent relationship. Circle size indicates the size of statistically significant correlations. Blue color when positively correlated, red color when negatively correlated. X indicates no statistically significant correlation. T1 = inquiry time point 1 (recruitment), T2 = inquiry time point 2 (after video information), T3 = inquiry time point 3 (after deliberation), T4 = inquiry time point 4 (one month after the citizens’ assembly). (TIF) [file pone.0258869.s004.tif]

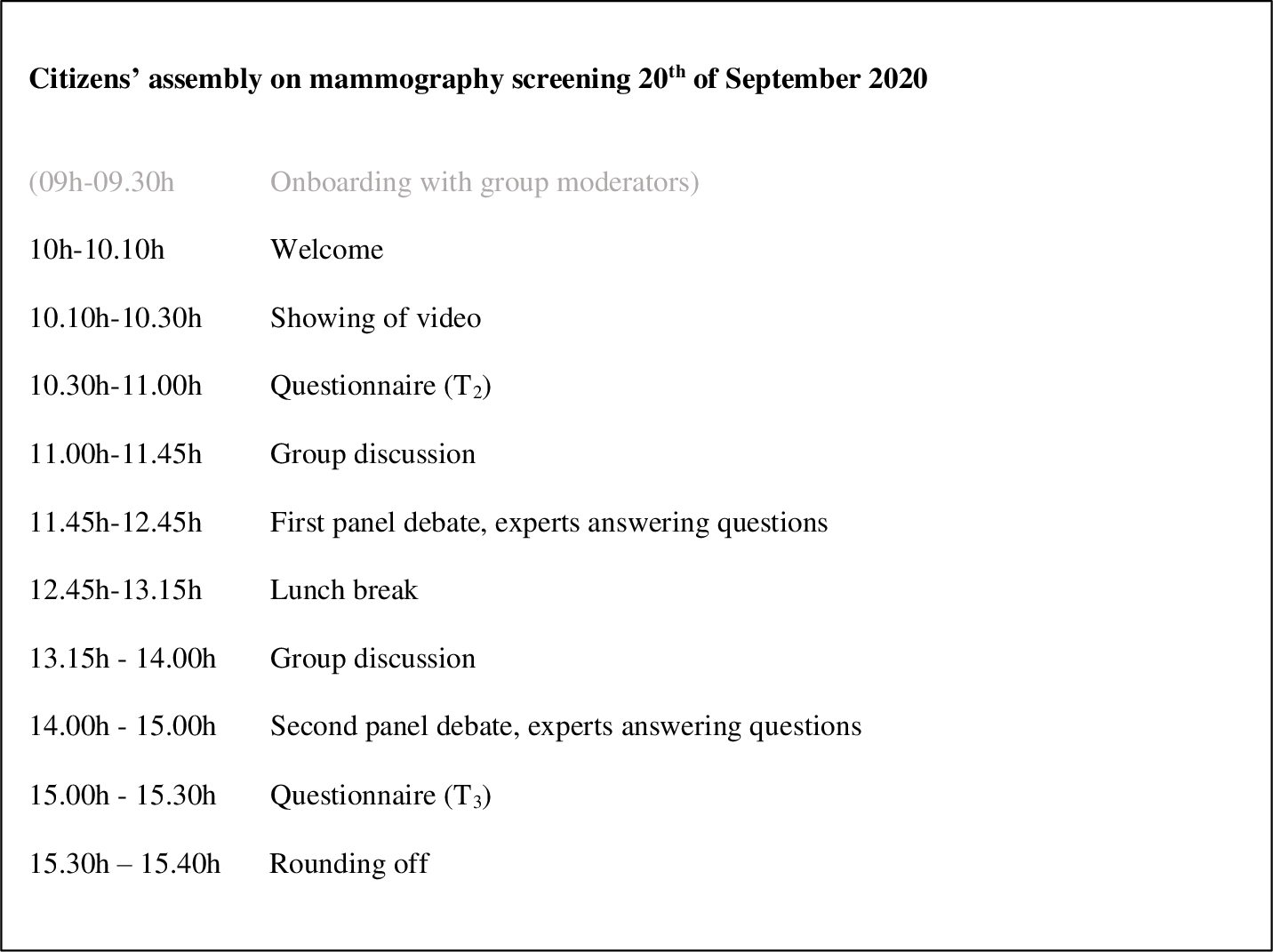

Supplement: S1 Table — (TIF) [file pone.0258869.s005.tif]

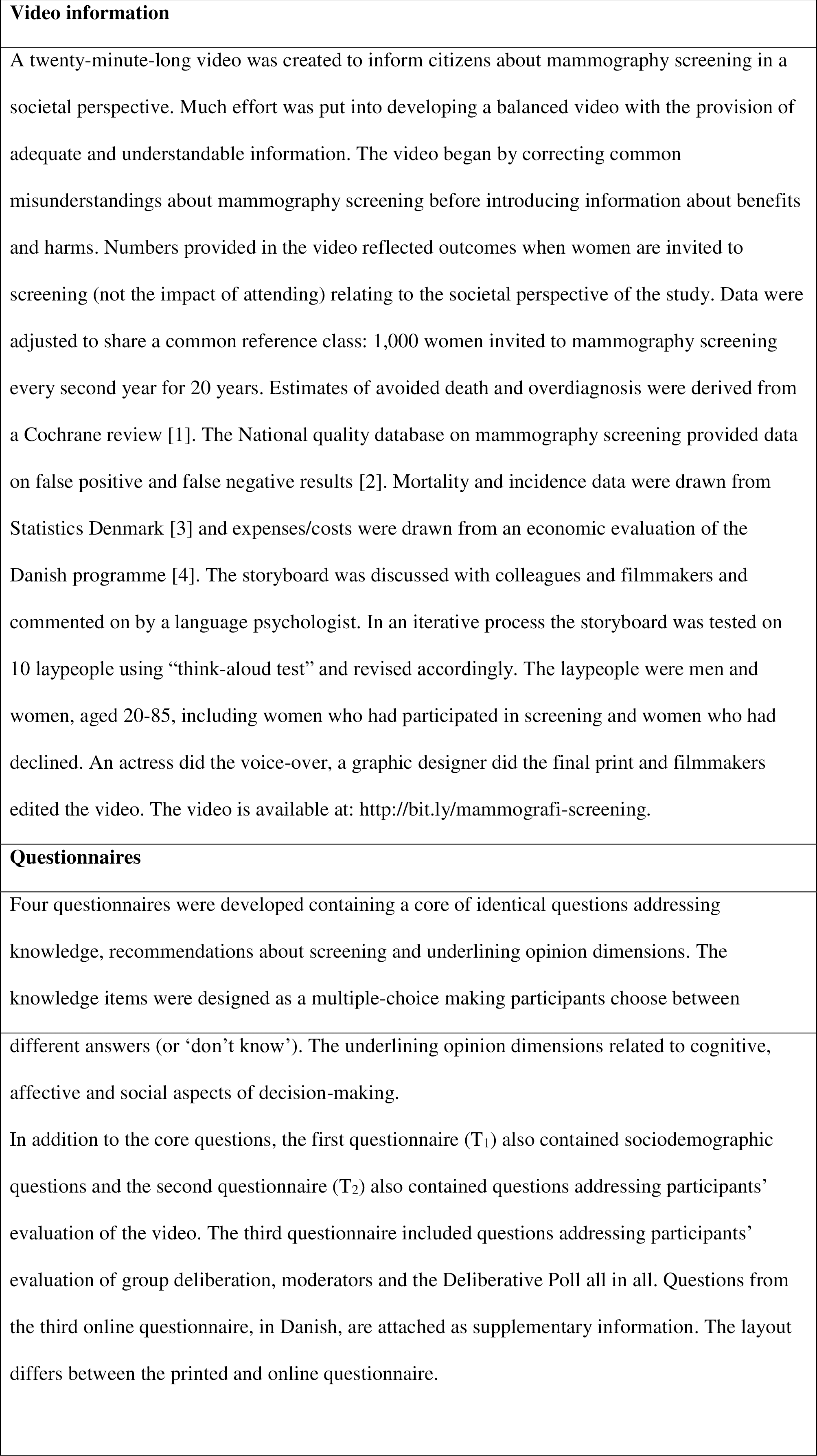

Supplement: S2 Table — (TIF) [file pone.0258869.s006.tif]

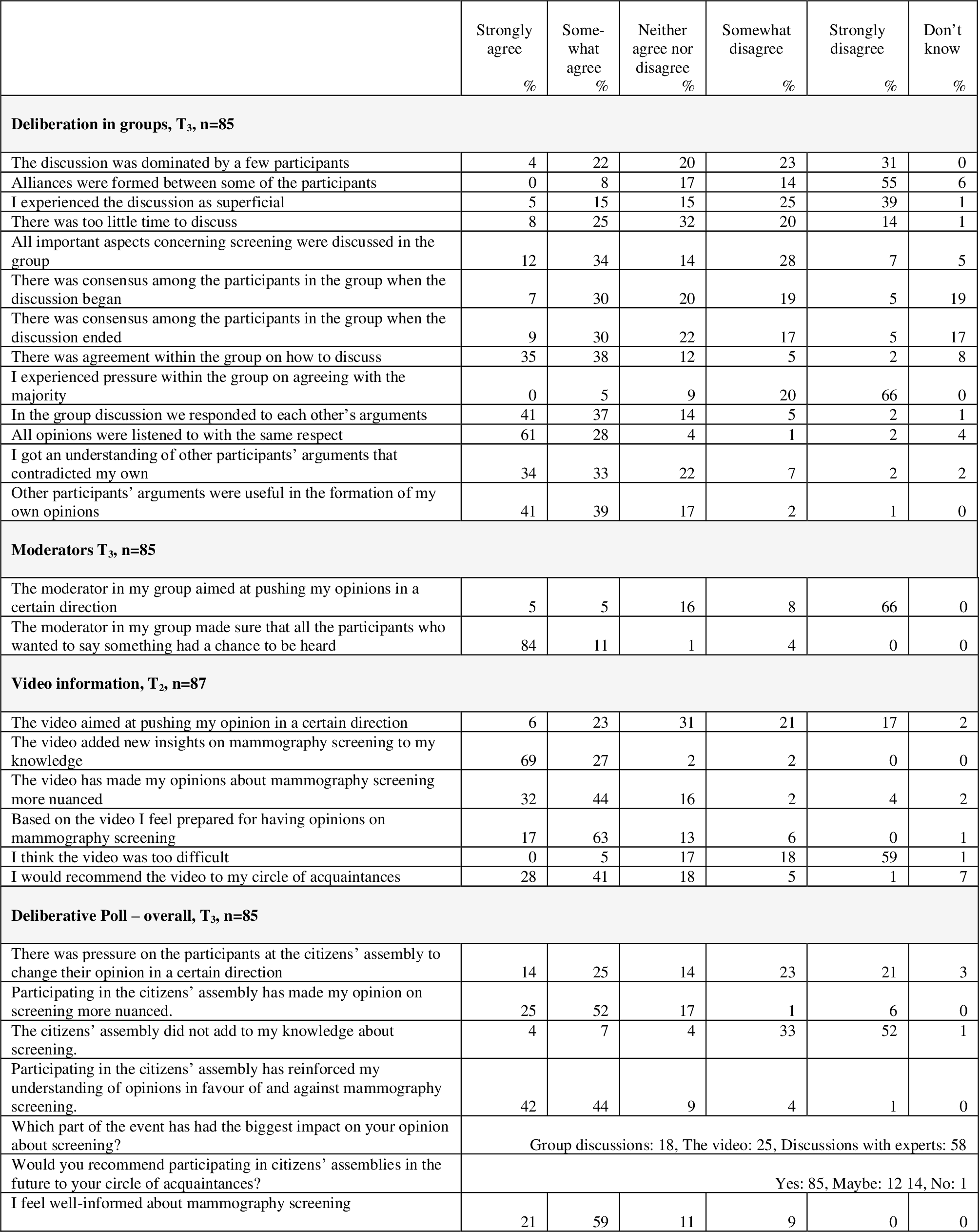

Supplement: S3 Table — Note: This table shows participants’ evaluation of group discussions, moderators, video information and the Deliberative Poll overall. T2 = inquiry time point 2 (after video information), T3 = inquiry time point 3 (after deliberation). (TIF) [file pone.0258869.s007.tif]

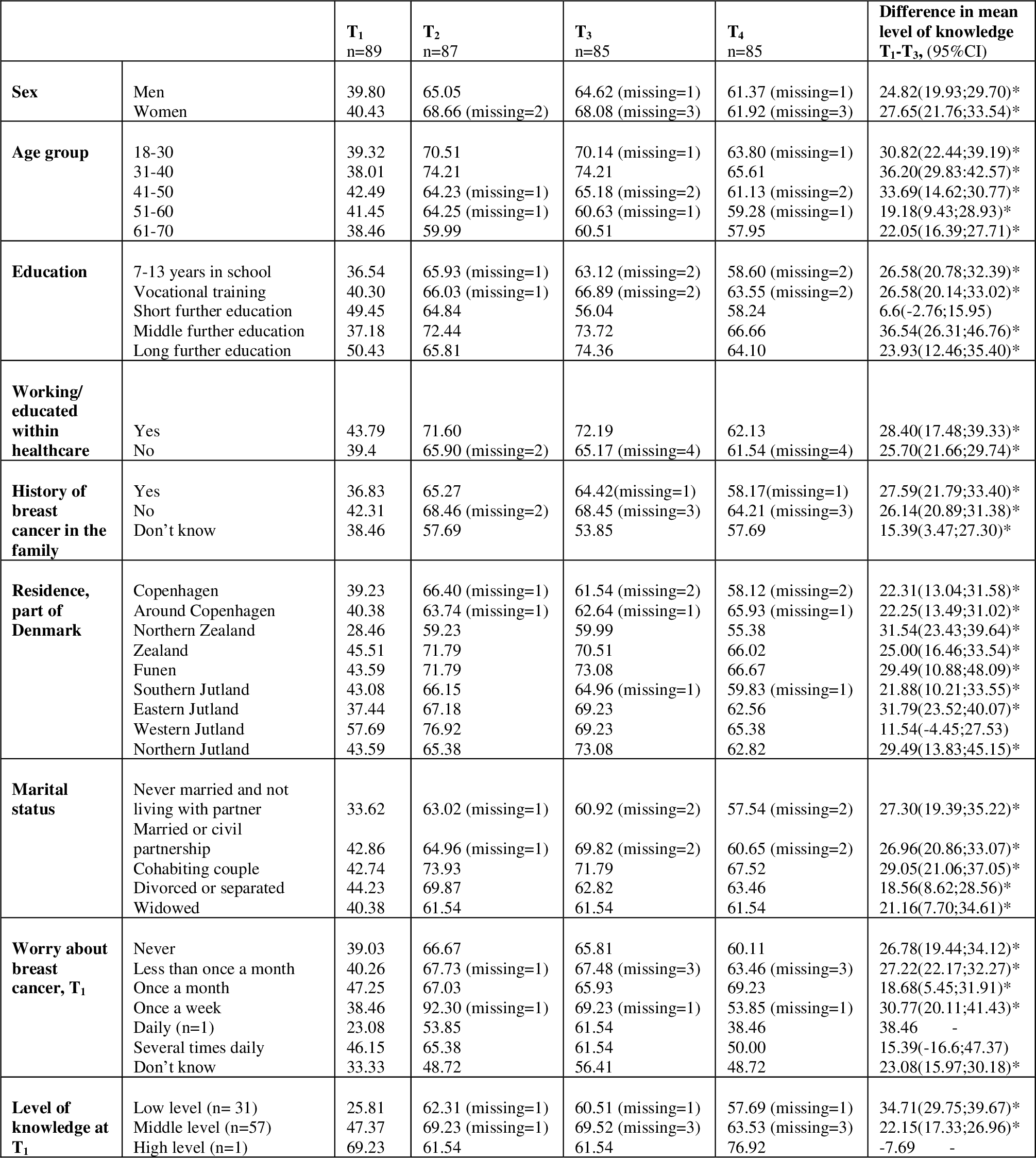

Supplement: S4 Table — Note: The knowledge index combines all 13 questions giving 7.69 points for each correct answer. The index ranges from 0 to 100 where 100 indicates correct answers to all questions and 0 incorrect answers to all questions. n varies between timepoints. T1 = inquiry time point 1 (recruitment), T2 = inquiry time point 2 (after video information), T3 = inquiry time point 3 (after deliberation), T4 = inquiry time point 4 (one month after the citizens’ assembly). (TIF) [file pone.0258869.s008.tif]

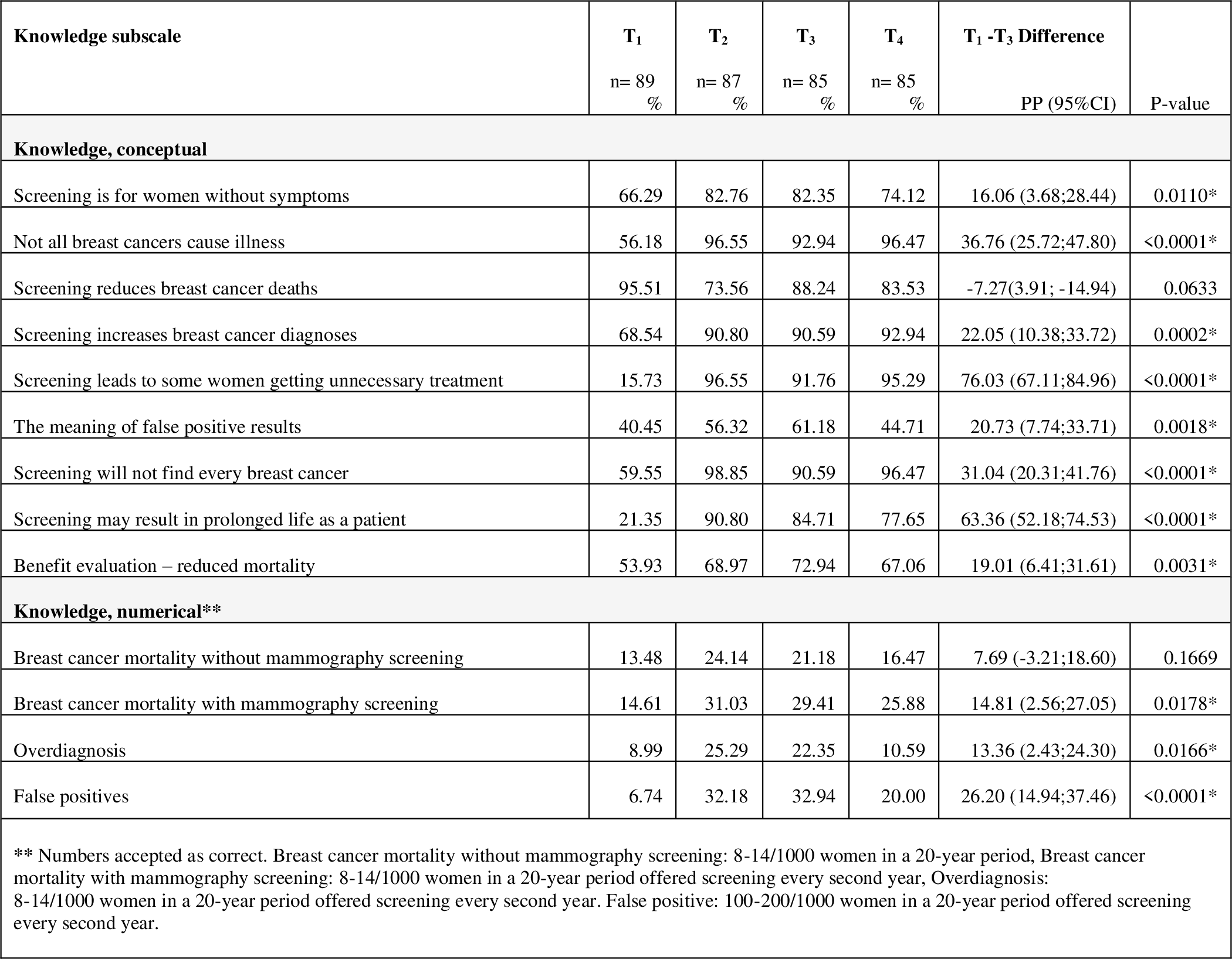

Supplement: S5 Table — Note: The table shows the level of knowledge at the four poll inquiry time points expressed as % correct answers to the 13 knowledge items. T1 = inquiry time point 1 (recruitment), T2 = inquiry time point 2 (after video information), T3 = inquiry time point 3 (after deliberation), T4 = inquiry time point 4 (one month after the citizens’ assembly). (TIF) [file pone.0258869.s009.tif]

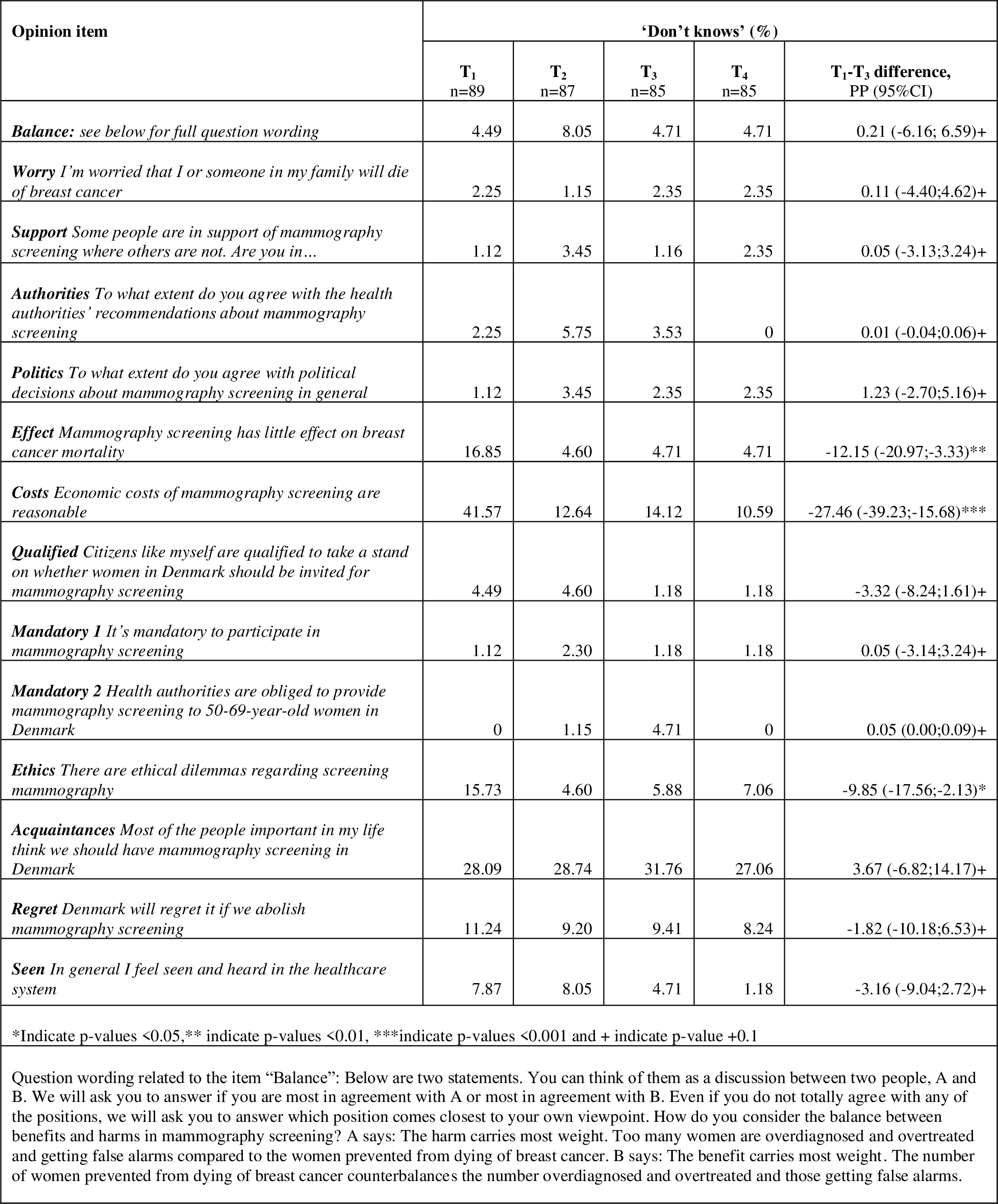

Supplement: S6 Table — Note: The table show the percentage of participants not able to form an opinion (answering ‘don’t know’) to each of the 14 opinion items. T1 = inquiry time point 1 (recruitment), T2 = inquiry time point 2 (after video information), T3 = inquiry time point 3 (after deliberation), T4 = inquiry time point 4 (one month after the citizens’ assembly). (TIF) [file pone.0258869.s010.tif]

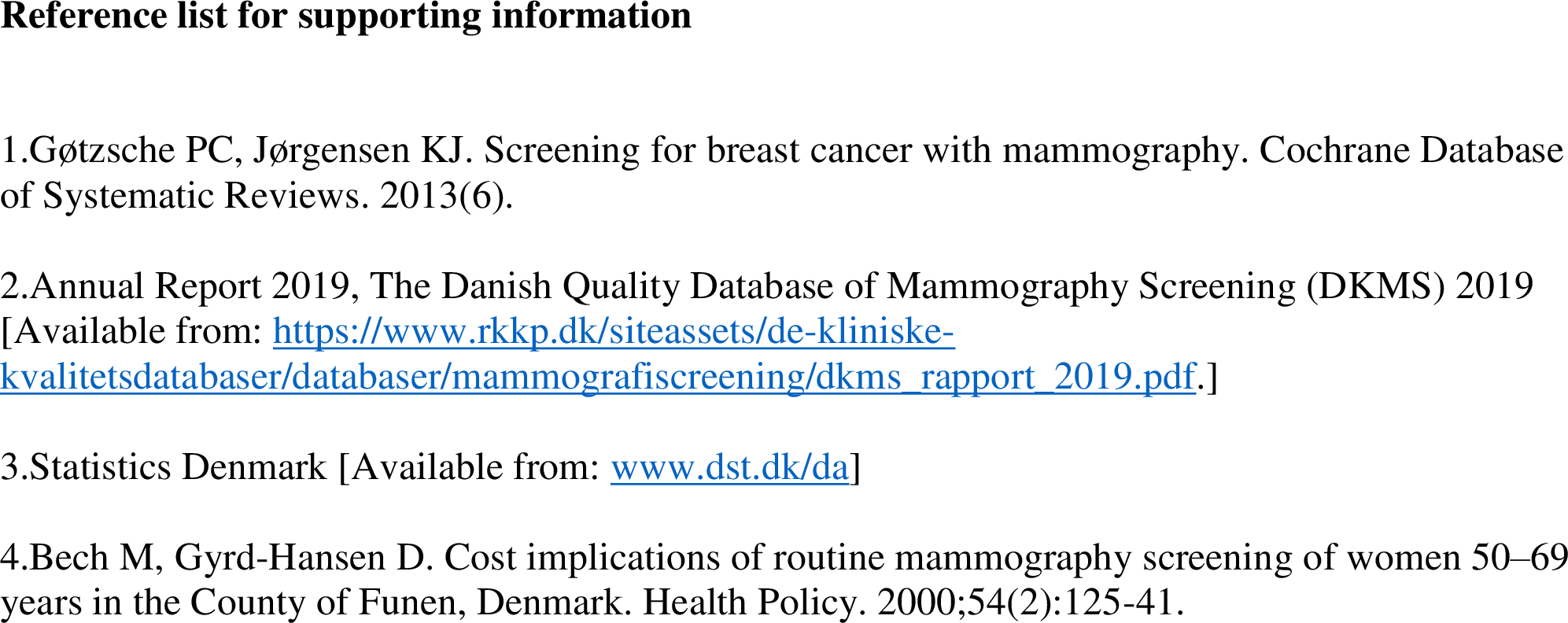

Supplement: S7 Table — (TIF) [file pone.0258869.s011.tif]
